# Post-transcriptional microRNA repression of *PMP22* dose in severe Charcot-Marie-Tooth disease type 1

**DOI:** 10.1093/brain/awad203

**Published:** 2023-06-20

**Authors:** Menelaos Pipis, Seongsik Won, Roy Poh, Stephanie Efthymiou, James M Polke, Mariola Skorupinska, Julian Blake, Alexander M Rossor, John J Moran, Pinki Munot, Francesco Muntoni, Matilde Laura, John Svaren, Mary M Reilly

**Affiliations:** Centre for Neuromuscular Diseases, Department of Neuromuscular Diseases, UCL Queen Square Institute of Neurology, London WC1N 3BG, UK; Waisman Center and Department of Comparative Biosciences, University of Wisconsin, Madison, WI 53706, USA; Centre for Neuromuscular Diseases, Department of Neuromuscular Diseases, UCL Queen Square Institute of Neurology, London WC1N 3BG, UK; Centre for Neuromuscular Diseases, Department of Neuromuscular Diseases, UCL Queen Square Institute of Neurology, London WC1N 3BG, UK; Centre for Neuromuscular Diseases, Department of Neuromuscular Diseases, UCL Queen Square Institute of Neurology, London WC1N 3BG, UK; Centre for Neuromuscular Diseases, Department of Neuromuscular Diseases, UCL Queen Square Institute of Neurology, London WC1N 3BG, UK; Centre for Neuromuscular Diseases, Department of Neuromuscular Diseases, UCL Queen Square Institute of Neurology, London WC1N 3BG, UK; Department of Clinical Neurophysiology, Norfolk and Norwich University Hospital, Norwich NR4 7UY, UK; Centre for Neuromuscular Diseases, Department of Neuromuscular Diseases, UCL Queen Square Institute of Neurology, London WC1N 3BG, UK; Waisman Center and Department of Comparative Biosciences, University of Wisconsin, Madison, WI 53706, USA; Dubowitz Neuromuscular Centre, NIHR Biomedical Research Centre at UCL Great Ormond Street Institute of Child Health and Great Ormond Street Hospital, London WC1N 1EH, UK; Dubowitz Neuromuscular Centre, NIHR Biomedical Research Centre at UCL Great Ormond Street Institute of Child Health and Great Ormond Street Hospital, London WC1N 1EH, UK; Centre for Neuromuscular Diseases, Department of Neuromuscular Diseases, UCL Queen Square Institute of Neurology, London WC1N 3BG, UK; Waisman Center and Department of Comparative Biosciences, University of Wisconsin, Madison, WI 53706, USA; Centre for Neuromuscular Diseases, Department of Neuromuscular Diseases, UCL Queen Square Institute of Neurology, London WC1N 3BG, UK

**Keywords:** dosage-sensitive genes, microRNAs, post-transcriptional regulation, peripheral myelin protein 22-kD, Charcot-Marie-Tooth disease type 1A

## Abstract

Copy number variation (CNV) may lead to pathological traits, and Charcot-Marie-Tooth disease type 1A (CMT1A), the commonest inherited peripheral neuropathy, is due to a genomic duplication encompassing the dosage-sensitive *PMP22* gene. MicroRNAs act as repressors on post-transcriptional regulation of gene expression and in rodent models of CMT1A, overexpression of one such microRNA (miR-29a) has been shown to reduce the PMP22 transcript and protein level.

Here we present genomic and functional evidence, for the first time in a human CNV-associated phenotype, of the 3′ untranslated region (3′-UTR)-mediated role of microRNA repression on gene expression.

The proband of the family presented with an early-onset, severe sensorimotor demyelinating neuropathy and harboured a novel *de novo* deletion in the *PMP22* 3′-UTR. The deletion is predicted to include the miR-29a seed binding site and transcript analysis of dermal myelinated nerve fibres using a novel platform, revealed a marked increase in *PMP22* transcript levels. Functional evidence from Schwann cell lines harbouring the wild-type and mutant 3′-UTR showed significantly increased reporter assay activity in the latter, which was not ameliorated by overexpression of a miR-29a mimic.

This shows the importance of miR-29a in regulating *PMP22* expression and opens an avenue for therapeutic drug development.

## Teaser

CMT1A is the commonest form of inherited neuropathy, presenting early in life with slowly progressive distal weakness and sensory loss, and is caused by duplication of the *PMP22* gene. Pipis *et al*. indirectly show that the microRNA miR-29a represses *PMP22* expression, and that loss of this effect can lead to more severe disease.

## Introduction

Segmental duplications are highly homologous genomic regions that seem to be major catalysts for the occurrence of copy number variants (CNVs), via recurrent non-allelic homologous recombination.^1^ CNVs are abundant in the general population, affecting up to 9.5% of the human genome.^1-3^ CNVs can account for significant non-pathological variation or lead to pathological phenotypes when they involve dosage-sensitive genes.^4^ With regards to rare neurological disorders, such CNVs are frequently observed in patients with intellectual disability with or without dysmorphisms,^5,6^ and in neurodegenerative diseases such as Charcot-Marie-Tooth disease (CMT)^7,8^ and, less so, Alzheimer’s disease.^9^

CMT1A is the commonest inherited peripheral neuropathy, with a population prevalence of ∼1:4200,^10^ and accounts for ∼60% of all genetically diagnosed cases of CMT. It is characterized by distal weakness and sensory loss with onset in the first two decades of life and patients commonly retain ambulation in late adulthood. It is most commonly caused by a 1.5 Mb duplication at the 17p locus, which includes the dosage-sensitive *PMP22* gene; deletion of the same genomic region causes another inherited neuropathy, hereditary neuropathy with liability to pressure palsies (HNPP). *PMP22* encodes for the 22-kD peripheral myelin protein 22, a tetraspan membrane protein in peripheral myelin, and the spectrum of *PMP22*-related disorders (loss of one allele causes HNPP, and the presence of three alleles causes CMT1A) illustrates the importance of the correct PMP22 dosage in myelin homeostasis. Further evidence supporting the *PMP22* gene-dosage molecular mechanism comes both from rodent models and human data. Several lines of transgenic mice and rats with multiple copies of the mouse *Pmp22* or human *PMP22* gene develop a peripheral demyelinating neuropathy,^11-13^ the severity of which is proportional to transgene copy number. Moreover, affected patients with a triplication at the 17p locus (thus harbouring four functional alleles of the gene) first develop symptoms in early childhood and lose independent ambulation early, a phenotype much more severe than the one commonly seen in CMT1A.^14,15^ Furthermore, in a large family in which multiple affected individuals had either the 17p deletion (manifesting HNPP) or the 17p duplication (manifesting CMT1A), two unaffected (clinically and neurophysiologically) siblings had co-occurrence of the deleted allele and duplicated allele, thus yielding the gene dosage that would be expected in the wild-type state and supporting the gene-dosage pathomechanism hypothesis.^16^

MicroRNAs are non-coding small RNA molecules, approximately 22 nucleotides (nts) long that are complementary to the 3′ untranslated region (3′-UTR) of transcripts through seed-specific microRNA binding sites that promote repression of the transcript through the RNA-induced silencing complex (RISC).^17^ Following early *in vitro* evidence that the *PMP22* 3′-UTR may have an inhibitory effect on gene expression,^18^ work by Verrier and colleagues^19^ showed the post-transcriptional regulatory effect of a specific microRNA (miR-29a) on endogenous *Pmp22* in cultured rat Schwann cells through a specific microRNA seed binding region in the transcript 3′-UTR; overexpression of miR-29a correlated with a reduction in the steady-state *Pmp22* transcript and protein levels.

Here, we present evidence of 3′-UTR-mediated post-transcriptional gene regulation, for the first time to our knowledge in humans, resulting in a well described CNV-associated clinical phenotype.

## Materials and methods

### Ethical approval and clinical data

Patients in this study were enrolled in the natural history protocol, which gained ethical approval from the Health Research Authority Research Ethics Committee (REC 09/H0716/61), and the 100 000 Genomes Project. All participants signed the relevant consent forms. Clinical data were collected prospectively during annual clinic visits and antecedent data were collected retrospectively from the patient history. The CMT Neuropathy Score version 2 (CMTNSv2) is a composite score (maximum 36) comprising nine items from the patients’ symptoms, examination findings and neurophysiological studies. The CMT Examination Score version 2 (CMTESv2) is a subscore (maximum 28) that only includes the patient’s symptoms and examination findings (seven items). A higher score indicates a higher level of impairment.

### Skin biopsy

Following appropriate written consent and under local anaesthetic (lidocaine 2%), two 3 mm punch skin biopsy samples were taken from the proband, from the ulnar aspect of the non-dominant forearm, 9 cm proximal to the ulnar crease. The biopsy samples were immediately submerged in two separate sterile, DNase-, RNase-, human and bacterial DNA-free, tubes (Eppendorf) each containing 2.1 ml of RNAlater solution (Qiagen) and frozen within 5 min of collection at −80°C.

### Whole exome and whole genome sequencing on whole blood

Whole exome sequencing (WES) was performed using an Agilent SureSelct V6 library on an Illumina HiSeq X platform. Whole genome sequencing (WGS) was performed using a TruSeq DNA PCR-free library on either HiSeq2000 or HiSeq X platforms at the Genomics England high-throughput facility (Hinxton, UK) as part of the 100 000 Genomes Project. Sequence reads were aligned to the human reference genome (UCSC GRCh37 for WES, UCSC GRCh38.p13 for WGS) and interrogated in relation to the coding exons of the major *PMP22* transcripts in Schwann cells (ENST0000312280 and ENST00000395938). Genomic loci were visually inspected for any structural variants using the Integrative Genomics Viewer (IGV).

### PCR amplicons gel electrophoresis and breakpoint junction delineation

Primers flanking the entire 650 bp deletion (A, B) and the telomeric and centromeric breakpoint junctions (C, D) were designed using Primer3 (https://primer3.ut.ee/) and SNPCheck V3 (https://genetools.org/SNPCheck/snpcheck.htm). For PCR amplification, the Phusion High-Fidelity DNA polymerase (New England Biolabs) was used in a touchdown temperature thermocycling PCR protocol. PCR products were visualized on a 2% agarose gel containing 10 μl GelRed dye for dsDNA staining. The PCR product bands were visualized under UV transilluminator, and digital photographs were taken using the Syngene GeneGenius image acquisition system and GeneSnap software (Synoptics). A GelPilot mid-range ladder (Cat. No. 239135; Qiagen) was used to visually verify the approximate size of the PCR amplicons. The relevant PCR product bands were extracted from the agarose gel and purified using the QIAquick Gel extraction and PCR and Gel Cleanup Kit (Qiagen). The purified PCR products underwent Sanger sequencing and the electropherograms were analysed to delineate the deletion’s precise breakpoint junctions.

### Nanostring transcript analysis

Total RNA was extracted from skin biopsies by homogenization/purification with the TRIzol® reagent, and then further purified using the Qiagen RNeasy MinElute Cleanup Kit (Cat. No. 74204). For the proband samples, the two punches were purified separately. The nCounter gene expression assay was performed by Nanostring (Seattle, WA) using the previously described custom Nanostring platform, as described.^20^ Nanostring data were analysed by nSolver Analysis Software v4.0. Raw counts were normalized to the geometric means of nSolver-selected normalization genes.

### Cloning of the 3′-UTR and reporter assays

The human *PMP22* 3′-UTR was cloned downstream of the luciferase gene into the pGL3 vector (Promega); the vector also contained the SV40 promoter. The exact coordinates of the human deletion were recreated in the del 3′-UTR version of this plasmid. The plasmids (100 ng) were transfected into the RT4 Schwann cell line (ATCC) and the Oli-neu oligodendrocyte cell line, along with 25 nmol of the hsa-miR-29a-3p mimic (Qiagen Cat. No. 219600) or control as indicated. The Promega Dual Luciferase Reporter Assay System and the Promega GloMax 20/20 luminometer was used to measure the luciferase activity of duplicate samples, which were normalized to the Renilla luciferase activity from a co-transfected pRL-TK plasmid (Promega) to control for transfection efficiency. Predicted microRNA’s targeting the *PMP22* 3′-UTR were identified by Targetscan.

## Results

**Figure 1:**
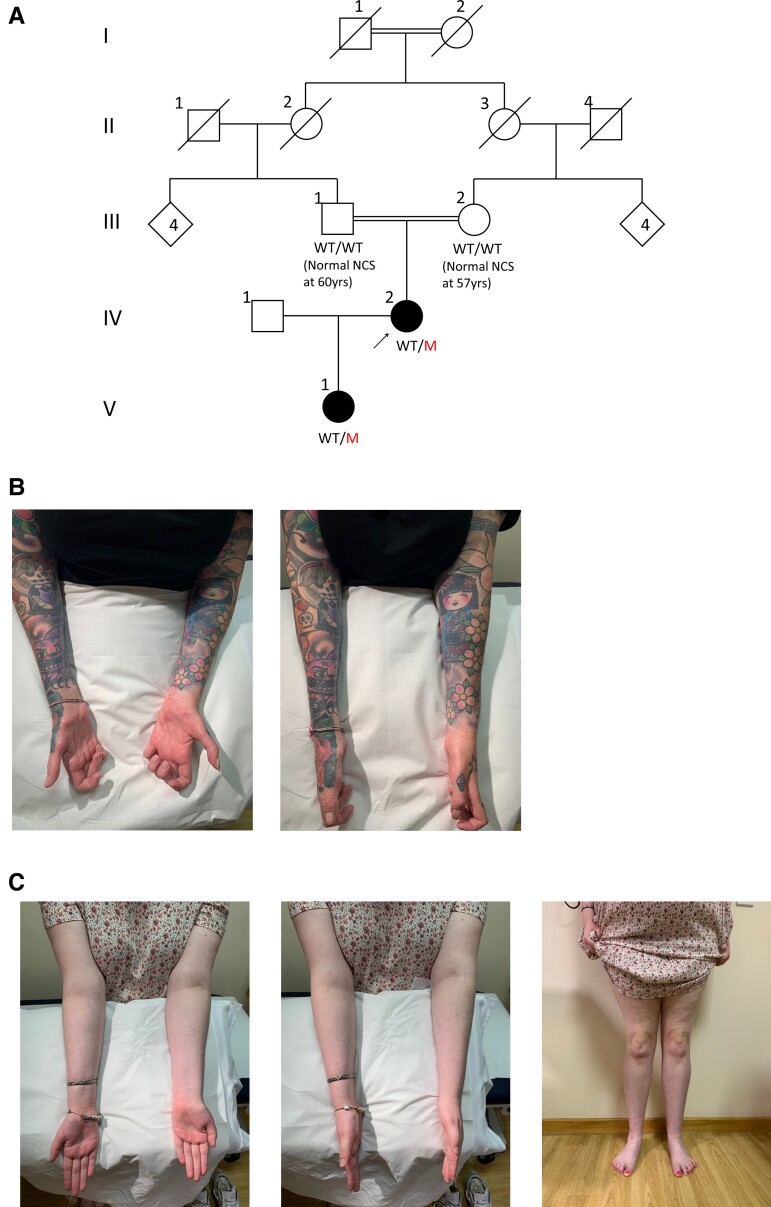
**Family pedigree and clinical photography of affected family members.** Family pedigree (**A**), with the arrow indicating the proband and filled symbols denoting affected individuals; squares are males and circles are females. In cases where the genotype was available, it is indicated under the corresponding symbol as WT/WT (wild-type/wild-type) or WT/M (wild-type/mutant). Still photographs showing forearm and intrinsic hand muscle amyotrophy in the proband (**B**) and a milder degree of forearm, hand and distal leg amyotrophy in the proband’s daughter (**C**).

The proband walked at 13 months but with splayed feet and never acquired the ability to run or jump in childhood (family pedigree in Fig. 1A). She developed high arches at age 7 years, and her walking difficulties and distal leg sensory loss progressed in her teens requiring multiple foot operations for foot deformities. She developed progressive hand dexterity difficulties and weakness in her early 20s; by age 27 years she required two sticks to mobilize and needed a mobility scooter outdoors from her mid-30s. Examination at the age of 48 years revealed significant amyotrophy below the knees and elbows (Fig. 1B). Motor examination showed length-dependent severe weakness in the lower limbs and the intrinsic hand muscles (Supplementary Table 1). She was areflexic throughout and had reduction in pinprick sensation up to the mid-forearms and knees and reduction in vibration sensation to the wrists and costal margins. The CMTNSv2 was 33 indicating severe disease. Neurophysiological studies at the age of 15 years revealed absent peroneal nerve compound muscle action potentials (CMAPs) and an ulnar motor nerve conduction velocity of 8.4 m/s. More recent studies at the age of 38 years revealed absent sensory nerve action potential (SNAPs) and CMAPs throughout and a very prolonged facial nerve motor latency (Supplementary Table 2).

The proband’s parents were first cousins and initially an autosomal recessive inheritance was suspected but her daughter presented with frequent tripping and multiple falls by the age of 3 years, having walked at 13 months, suggesting an autosomal dominant inheritance. The proband’s daughter experienced difficulties running as a child and developed walking difficulties in her early teens and frequent cramps when writing, evocative of early denervation. Examination at the age of 17 years showed mild amyotrophy in the hands and below the knees (Fig. 1C), toe clawing and a subtle foot drop when walking. Motor examination revealed mild distal weakness in the upper and lower limbs (intrinsic hand muscles 4/5, ankle dorsiflexion 4−/5, ankle plantarflexion 4+/5). She was areflexic and pinprick sensation was reduced to above the toes. CMTESv2 was 10 indicating mild-moderate disease. Neurophysiological studies at age 8 years revealed absent sensory responses and reduced motor responses with severe slowing in motor conduction velocities, consistent with the mother’s demyelinating phenotype (Supplementary Table 2).

**Figure 2:**
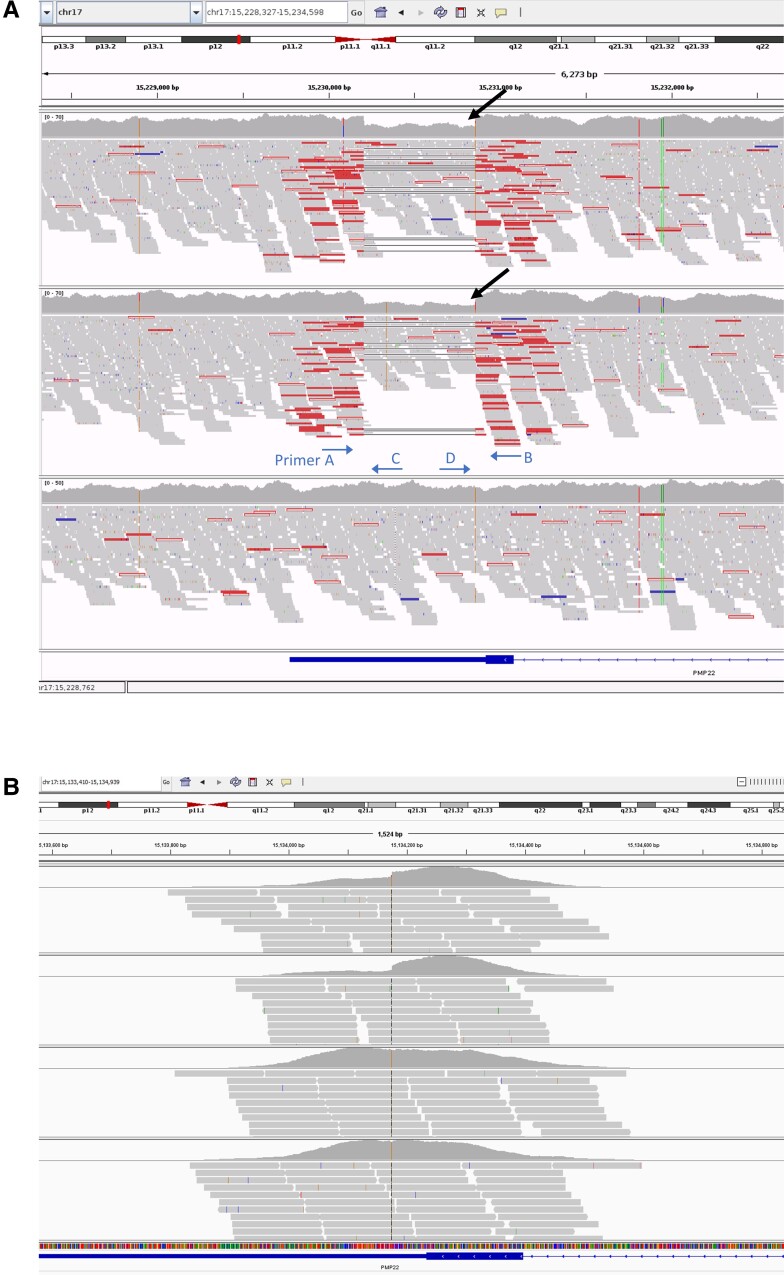
**Aligned sequence reads at the *PMP22* locus visualized in the IGV browser.** Sequence reads from the trio WGS study (**A**) and the quad WES study (**B**). In the WGS study, the panels correspond to the proband (*top*), proband’s daughter (*middle*) and the proband’s mother (*bottom*) and the black arrows indicate the 50% reduction in the coverage plot. The approximate primer annealing sites and direction are indicated. In the WES study (panel order from *top* to *bottom* is proband, proband’s daughter, proband’s mother, proband’s father), there is a hint of reduced coverage at the *PMP22* 3′-UTR in the proband and the proband’s daughter, but is not as obvious and well delineated as in the WGS study, and hence could be overlooked. IGV = Integrative Genomics Viewer; WES = whole exome sequencing; WGS = whole genome sequencing.

**Figure 3:**
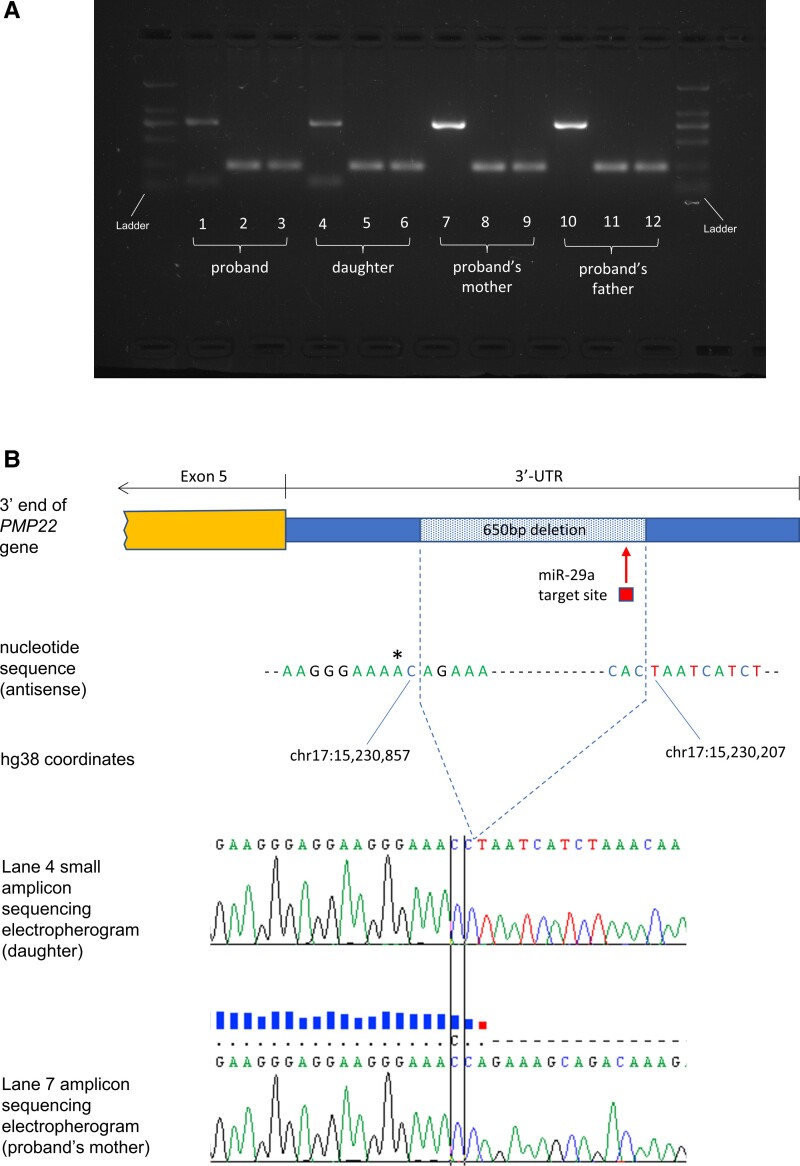
**Delineation of the breakpoint junctions of the structural variant.** Gel electrophoresis of PCR amplicons (**A**): primers A + B correspond to Lanes 1, 4, 7, 10, primers A + C correspond to Lanes 2, 5, 8, 11, and primers D + B correspond to Lanes 3, 6, 9 and 12. Lanes 1 and 4 illustrate the presence of both the wild-type PCR amplicon (779 bp) and mutated (129 bp). Lanes on either side of Lanes 1–12 were used for the mid-range ladder. Breakpoint junction sequencing was performed on the amplicons excised from Lanes 4 and 7 and the corresponding electropherograms are shown in **B** (midline of the electropherogram is centred on the SNP position indicated by an asterisk). The electropherogram from the small amplicon in Lane 4 containing the 3′-UTR deletion shows the interruption and skipping of the reference nucleotide sequence whereas the electropherogram from the amplicon in Lane 7 shows an uninterrupted reference sequence. The asterisk at genomic position chr17:15,230,858 corresponds to the common SNP rs13422 (MAF G = 0.32), which was present homozygously in both the proband’s parents as well as the proband and her daughter. The predicted miR-29a target site is also illustrated in respect to the *PMP22* 3′-UTR deletion.

Multiple genetic tests on whole blood were negative in the proband including multiplex ligation-dependent probe amplification for the 17p rearrangement, extensive next-generation sequencing CMT gene panels, quad WES and single nucleotide variant and indel analysis on WGS data. By visually inspecting the paired-end mates at the *PMP22* locus in the proband’s, proband’s daughter’s and proband’s mother’s WGS data, we identified a 650 bp deletion within the 3′-UTR of the gene with a corresponding 50% reduction in read depth in the proband and the daughter, suggesting the deletion was restricted to one allele and inherited (Fig. 2). The variant was absent, apart from our two patients in this study, from the 100 000 Genomes Project database (which has a large number of disease controls, including other rare non-neurological diseases as well as cancer germline genomes) and from gnomAD SVs v2.1. Using PCR amplification across the deletion locus and gel electrophoresis of the amplicons, we confirmed the presence of the deletion in the heterozygous state in the proband and the daughter and delineated the breakpoint junctions (small fragment from Lanes 1 and 4 in Fig. 3A).

**Figure 4:**
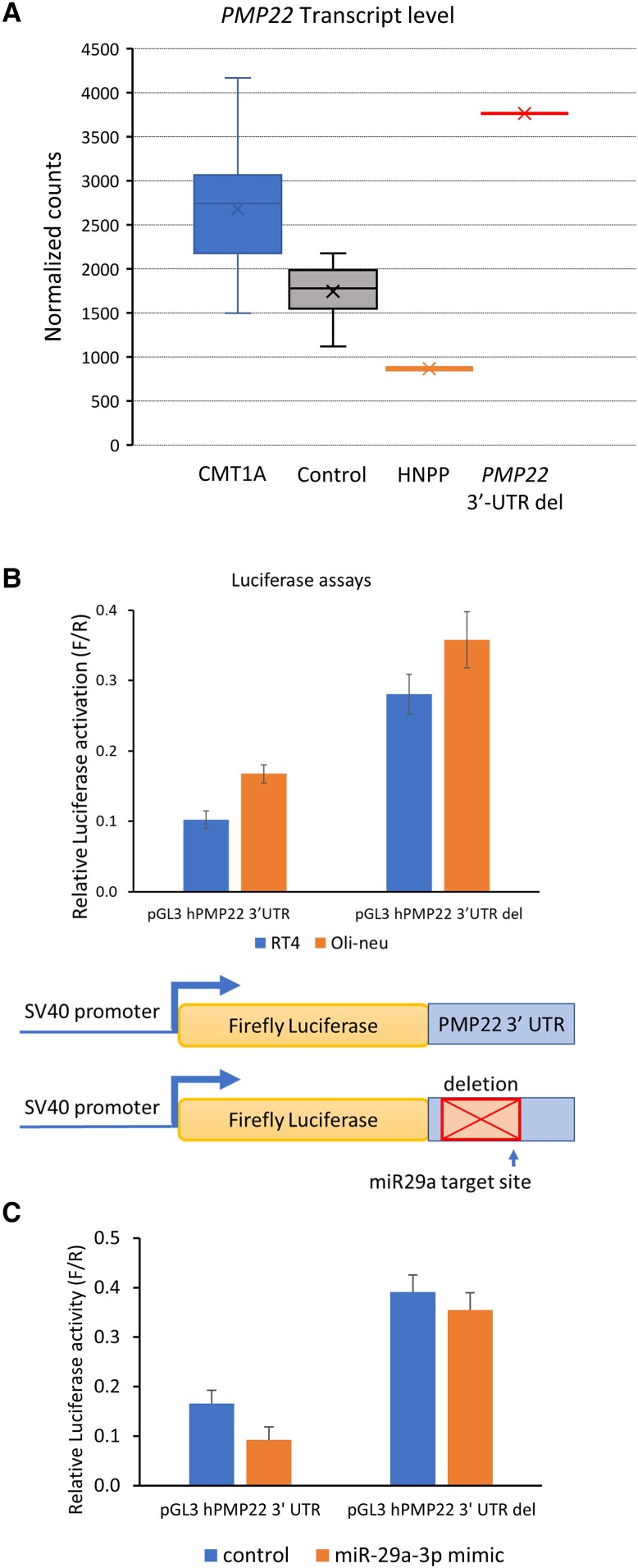
**The 3**′**-UTR deletion elevates *PMP22* transcript levels.** (**A**) For skin biopsy samples from controls (*n* = 16), HNPP (*n* = 1) and CMT1A patients (*n* = 15) and the proband (from two duplicate skin biopsies) in this study, purified RNA was submitted for Nanostring transcript quantitation analysis. The box and whisker plot shows *PMP22* transcript values for the samples after normalization to Schwann cell-specific genes. (**B**) The graph shows normalized reporter activity from luciferase plasmids containing the human *PMP22* 3′-UTR or a version in which the neuropathy-associated deletion was introduced (3′-UTR del). The transfections were performed in the RT4 Schwann cell and Oli-neu oligodendrocyte cell lines. Error bars indicate standard deviations. (**C**) The two reporter plasmids were transfected with a control siRNA or a miR-29a mimic in RT4 cells, and resulting luciferase activities indicate that deletion of the 3′-UTR renders the luciferase reporter resistant to miR-29a repression. Reduction of luciferase activity by miR-29a co-transfection was significant (*P* < 0.02, *t*-test, *n* = 3) for the wild-type construct. HNNP = hereditary neuropathy with liability to pressure palsies.

Since the affected family members had a more severe demyelinating CMT phenotype compared to CMT1A we performed transcript quantitation analysis to quantify the *PMP22* transcript levels in skin biopsy samples containing Schwann cells from epidermal myelinated nerve fibres, using a custom Nanostring platform that was developed to detect elevated levels of *PMP22* in CMT1A.^20^ After standardizing transcript counts relative to Schwann cell-specific normalizing genes, the *PMP22* transcript levels in duplicate skin biopsies were markedly increased compared to known controls and disease controls (CMT1A). *PMP22* levels in HNPP were reduced ∼50%, as would be predicted (Fig. 4A). Using the TargetScan *in silico* prediction tool, we ascertained that the miR-29a binding site falls within the 3′-UTR deletion coordinates in the human genome (Fig. 3B). Thereafter, we cloned the 3′-UTR with and without the deletion into a pGL3-promoter luciferase vector, transfected it into RT4 Schwann cell-like and Oli-neu oligodendrocyte lines and found elevated luciferase reporter activity from the construct with the 3′-UTR deletion, suggesting loss of a repressor effect on gene expression (Fig. 4B). As a complementary approach, we overexpressed a miR-29a mimic and found that 3′-UTR deletion rendered the luciferase reporter resistant to miR-29a repression (Fig. 4C).

## Discussion

In this study, we illustrate for the first time how imbalance in the microRNA-mediated regulation of gene expression can mimic a CNV-associated disease phenotype in humans. This was possible because of the unique co-occurrence of a 3′-UTR deletion disrupting the microRNA-mediated repression of the dosage-sensitive *PMP22* gene, which has a well characterized overexpression (CMT1A) and under-expression (HNPP) phenotype caused by duplication and deletion, respectively. This study complements earlier *in vitro* evidence of the 3′-UTR repressor role in rat and human *PMP22* expression.^18,19,21-23^ While *PMP22* is one of the most highly expressed transcripts in myelinating Schwann cells, it is also clear that its expression needs to be maintained within a narrow range.

Our phenotype-genotype-functional study correlation indirectly confirmed the repression exerted by miR-29a on the 3′-UTR of the *PMP22* transcript although other microRNAs may also be involved. Loss-of-function studies of miR-29 family members have not yet been performed *in vivo*, but seed binding sites for miRNAs 139/145/199 are predicted (TargetScan 8.0) to fall within the 3′-UTR deletion harboured by affected members of this family, and may also play a role in the repression of *PMP22* expression. Interestingly, assuming equal allelic expression, the loss of microRNA repression in one allele (since the deletion was in the heterozygous state) caused higher *PMP22* transcript counts in dermal Schwann cells compared to transcript counts from CMT1A disease controls. This is entirely in keeping with the more severe demyelinating CMT phenotype observed in our proband, compared to the natural history of CMT1A,^24^ and is akin to the phenotype expressed by patients carrying a *PMP22* triplication.^14^ The relationship between gene dosage (and by extension protein concentration) and expressed phenotype is complex and largely influenced by the biological function of the protein of interest.^4^ In CMT1A, both *PMP22* mRNA and protein have been shown to be elevated in dermal nerve, although there is substantial variation in these levels.^20,25^

The role of microRNAs in regulating *PMP22* expression is supported by multiple lines of evidence. *In vitro* evidence from co-transfection studies shows that overexpression of miR-9 represses *Pmp22-*reporter activity in HeLa cells^21^ and overexpression of miR-29a reduces endogenous *PMP22* steady-state transcript levels in rat Schwann cells^19^ and dermal fibroblasts from CMT1A patients.^23^ Furthermore, an *in vivo* study of intraneural administration of a miR-381 expressing lentiviral vector in sciatic nerves of C22 mice, a well characterized CMT1A mouse model, shows amelioration of the phenotype and improved sciatic nerve myelination.^22^ Disease onset and severity in CMT1A is remarkably heterogeneous, despite most affected individuals carrying the same 1.5 Mb 17p duplication and microRNAs have been contemplated as potential genetic modifiers of the phenotype. Using a candidate gene hypothesis, a case-control sequencing association study showed a significant association between the genotypes of a miR-149 SNP and disease onset and severity in CMT1A,^26^ but a case-only replication study in an independent CMT1A cohort failed to support this association, which may have been due to a small sample size.^27^

It is also important to highlight the fidelity of PCR-free whole genome sequencing in identifying such small structural variants, especially in the non-coding genome where other genetic methods, such as WES may not detect it due to enrichment and sequence bias, and microarray studies due to the small size of the structural variant. *De novo* non-recurrent deletions, as in this case, are most likely to have a germline origin and arise by replication-based mechanisms such as microhomology-mediated break-induce repair.^28^

In the current era of genetic therapy development, the exploration of 3′-UTR-mediated microRNA repression of gene expression also opens another avenue for therapeutic drug development. Recent studies have shown that antisense oligonucleotides and RNAi approaches targeting the *PMP22* transcript are effective in rodent models of CMT1A.^29,30^ As cellular studies have already shown that overexpression of miR-29a in dermal fibroblasts derived from CMT1A patients downregulates human *PMP22* expression,^23^ this may prove to be an attractive therapeutic target in a dosage-sensitive disease.

## Supplementary Material

awad203_Supplementary_Data

## Data Availability

The data that support the findings of this study are available from the corresponding author, upon reasonable request. The data are not publicly available since they contain information that could compromise the privacy of research participants.
