# Supplementary material for: Post-transcriptional microRNA repression of PMP22 dose in severe Charcot-Marie-Tooth disease type 1
Source: Brain. 2023 Jun 20;146(10):4025–32. doi: 10.1093/brain/awad203 (PMC10545524; doi:10.1093/brain/awad203)
Supplement: awad203_Supplementary_Data [file awad203_supplementary_data.pdf]

Supplementary Tables.

Supplementary Table 1

Muscle strength (MRC grade)	Right	Left
**Upper limbs**		
Shoulder abduction	5	4
Elbow flexion	4+	4
Elbow extension	5	5
Wrist extension	4	4
Finger extension	4	4
FDIO	0	0
ADM	0	0
APB	0	0
**Lower limbs**		
Hip flexion	4	4
Hip extension	5	5
Knee flexion	1	3
Knee extension	5	5
Ankle dorsiflexion	0	0
Ankle plantarflexion	0	0

Clinical examination of muscle strength in proband age 48 years. All scores are on the MRC grading scale. Abbreviations: ADM, abductor digiti minimi; APB, abductor pollicis brevis; FDIO, first dorsal interosseous.

Supplementary Table 2

**Neurophysiological studies**		**Proband (IV.2)**			**Daughter (V.1)**	
**Age at assessment**	38 years			8 years		
**Sensory studies**	Lat (ms)	SNAP (μV)	CV (m/s)	Lat (ms)	SNAP (μV)	CV (m/s)
Median nerve (palm – wrist)		Absent		-	Absent	-
Radial nerve (forearm – wrist)		Absent			-	
Superficial peroneal (calf – med. dors. cutan.)		-		-	Absent	-
**Motor studies**	Lat (ms)	CMAP (mV)	CV (m/s)	Lat (ms)	CMAP (mV)	CV (m/s)
Peroneal nerve (ankle – EDB)		-		10.5	0.46	
Peroneal nerve (below knee – ankle)		-			0.29	9.5
Ulnar nerve (wrist – ADM)		Absent		7.5	4.0	
Ulnar nerve (above elbow – wrist)		-			1.85	12.4
Median nerve (wrist – APB)		Absent			-	
Facial nerve (nasalis)	13.4	0.2			-	
**EMG comments**	Widespread and severe chronic denervation in orbicularis oculi, biceps and vastus medialis			Moderate chronic denervation in right tibialis anterior		

Summary of neurophysiological studies. All motor amplitudes from the limbs are responses to distal stimulation (wrist and ankle). Abbreviations: -, not available; ADM, abductor digiti minimi; APB, abductor pollicis brevis; CMAP, compound muscle action potential; CV, conduction velocity; EDB, extensor digitorum brevis; EMG, electromyography; Lat, latency; SNAP, sensory nerve action potential.
